# Effects of physical exercise on natural killer cell activity during (neo)adjuvant chemotherapy: A randomized pilot study

**DOI:** 10.14814/phy2.14919

**Published:** 2021-06-10

**Authors:** Elisa C. Toffoli, Maike G. Sweegers, Hetty J. Bontkes, Teatske M. Altenburg, Henk M.W. Verheul, Hans J. van der Vliet, Tanja D. de Gruijl, Laurien M. Buffart

**Affiliations:** ^1^ Department of Medical Oncology Amsterdam UMC Vrije Universiteit Amsterdam Amsterdam The Netherlands; ^2^ Department of Epidemiology and Biostatistics Amsterdam UMC Vrije Universiteit Amsterdam Amsterdam Public Health Amsterdam The Netherlands; ^3^ Department of Clinical Chemistry Amsterdam UMC Vrije Universiteit Amsterdam Amsterdam Netherlands; ^4^ Department of Public and Occupational Health Amsterdam Public Health Research Institute Amsterdam UMC Vrije Universiteit Amsterdam Amsterdam The Netherlands; ^5^ Department of Medical Oncology Radboud University Medical Center Radboud Institute for Health Sciences Nijmegen The Netherlands; ^6^ Lava Therapeutics Utrecht Netherlands; ^7^ Department of Physiology Radboud University Medical Center Radboud Institute for Health Sciences Nijmegen The Netherlands

**Keywords:** cancer, chemotherapy, natural killer cells, NK cell activity, physical exercise

## Abstract

Natural killer (NK) cells are a population of innate immune cells known to play a pivotal role against tumor spread. In multiple murine models, it was shown that physical exercise had the potential to increase NK cell antitumor activity through their mobilization and tissue redistribution in an interleukin (IL)‐6 and epinephrine‐dependent manner. The translation of this finding to patients is unclear. In this randomized pilot trial, we analyzed blood samples of patients with resectable breast or colon cancer who were randomized into an evidence‐based moderate‐high intensity resistance and aerobic exercise intervention (*n* = 8) or a control group (*n* = 6) during the first 9–12 weeks of (neo)adjuvant chemotherapy. In this pilot, we did not solely focus on statistical significance, but also explored whether average between‐group differences reached 10%. NK cell degranulation was preserved in the exercise group whereas it decreased in the control group resulting in a between‐group difference of 11.4% CD107a^+^ degranulated NK cells (95%CI = 0.57;22.3, *p* = 0.04) in the presence and 13.8% (95%CI = −2.5;30.0, *p* = 0.09) in the absence of an anti‐epidermal growth factor receptor monoclonal antibody (EGFR‐mAb). In line, the between‐group difference of tumor cell lysis was 7.4% (95%CI = −9.1;23.9, *p* = 0.34), and 13.7% (95%CI = −10.1;37.5, *p* = 0.23) in favor of the exercise group in the presence or absence of EGFR mAb, respectively. Current explorative analyses showed that exercise during (neo)adjuvant chemotherapy may benefit NK cell activity. Future studies with a larger sample size are needed to confirm this finding and to establish its clinical potential.

Trial registration: Dutch trial register number NTR4105.

## Graphical abstract

We analyzed blood samples of patients with resectable breast or colon cancer who were randomized into an evidence‐based exercise intervention (*n* = 8) or control group (*n* = 6) during the first 9–12 weeks of (neo)adjuvant chemotherapy. NK cell degranulation was preserved during exercise and, although not statistically significant, cytotoxicity levels were 7%–14% higher compared to controls suggesting that physical exercise during (neo)adjuvant chemotherapy might benefit NK cell activity.

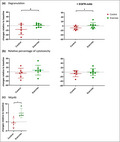

## 1 INTRODUCTION

Natural killer (NK) cells are innate immune cells that play a key role in the defense against tumor or virus‐infected cells. Their activity is regulated by a balance of activating and inhibitory receptors. The former comprises the natural killer group 2 member (NKG2)D/C/E, the natural cytotoxicity receptors (NCRs), such as NKp30/44/46, and the DNAX Accessory Molecule‐1 (DNAM1) while the latter includes the killer cell immunoglobulin‐like receptors (KIR)2D and NKG2A (Vivier et al., 2008). Human NK cells can be divided into two main subsets with distinct immune functions: the CD56^dim^CD16^+^ NK cells traditionally described as more cytotoxic and the CD56^brigth^CD16^low/neg^ characterized by the ability to produce high levels of cytokines (Cooper et al., 2001; Vivier et al., 2008). NK cell activity can be compromised by cancer and previous studies demonstrated that patients with cancer who have activated NK cells have a better prognosis (Takeuchi et al., 2001; Tartter et al., 1987). Activated NK cells can also improve the response to antibody‐based therapies (Beano et al., 2008; Liljefors et al., 2003). However, chemotherapy among other anti‐tumor therapies, can lead to reduced NK cell activity (Beitsch et al., 1994; Brenner & Margolese, 1991; Sewell et al., 1993) increasing the need for strategies to maintain it. Physical exercise plays a role in the regulation of the immune system and, potentially, in cancer control (Idorn & Hojman, 2016). In different mouse models, a direct inhibitory effect of exercise on tumor growth was found (Pedersen et al., 2016), where the exercise‐induced release of epinephrine and interleukin (IL)‐6 into the bloodstream resulted in mobilization, redistribution, and activation of NK cells, with increased infiltration of activated NK cells (Pedersen et al., 2016). It is unclear whether these findings can be translated to patients. Studies examining NK cell activation after exercise interventions in patients with cancer are scarce, and none were conducted during chemotherapy (Zimmer et al., 2017). With this pilot trial, we aimed to explore the effects of exercise on NK cell phenotype and function, and plasma IL‐6 levels during chemotherapy treatment.

## 2 METHODS

### 2.1 Design and participants

This study was part of the Mechanisms of Training In patients with Cancer (METRIC) a randomized multicenter pilot trial conducted in three enrolling sites in the Netherlands: Amsterdam UMC (location VU University Medical Center), Amstelland Hospital, and the Netherlands Cancer Institute/Antoni van Leeuwenhoek Hospital. Patients diagnosed with resectable colon (stage II/III) or breast cancer stage (I/II/III) scheduled to receive (neo)adjuvant chemotherapy were randomized into an evidence‐based moderate‐high intensity resistance and aerobic exercise intervention (Waart et al., 2015) during the first 9–12 weeks of chemotherapy or to a control group. Blood samples were obtained before the start of chemotherapy and randomization (T0), and after 9–12 weeks of chemotherapy (T1).

Patient exclusion criteria included (1) the inability to perform basic activities of daily living such as walking or cycling, (2) the presence of cognitive disorders or severe emotional instability, (3) the existence of other disabling co‐morbidities that hampered physical activity, (4) the inability to read Dutch, (5) concurrent or prior anticancer therapies, (6) prednisone therapy concurrent or up to 6 months before diagnosis, and (7) insulin dependency. This study was conducted according to the declaration of Helsinki and it was approved by the medical ethics committee (VUmc: 109 2012/430) and registered in the Dutch Trial Registry in 2013 (NL3944). All patients signed an informed consent form prior to participation in the study.

### 2.2 Exercise intervention and control group

The exercise intervention consisted of two weekly 60‐min exercise sessions that were supervised by a physiotherapist specifically educated to train patients with cancer. Each exercise session started with warming‐up exercises, followed by 20 min resistance exercise training and 30 min aerobic exercise training at moderate‐high intensity, and ended with a cooling down (Buffart et al., 2020). During the first appointment, maximal short exercise capacity and muscle strength were determined through a steep ramp test and indirect 1‐repetition maximum test, respectively. Afterward, these tests were repeated every three weeks to ensure adequate training intensity. Further details about the study procedures and exercise intervention are presented elsewhere (Buffart et al., 2020). Patients in the control group were offered the evidence‐based exercise intervention during the second half of the chemotherapy (after the T1 assessments) to decrease the number of drop‐outs due to disliking of the randomization and reduce non‐compliance (Steins Bisschop et al., 2015).

### 2.3 NK cell degranulation and cytotoxicity

**FIGURE 1:**
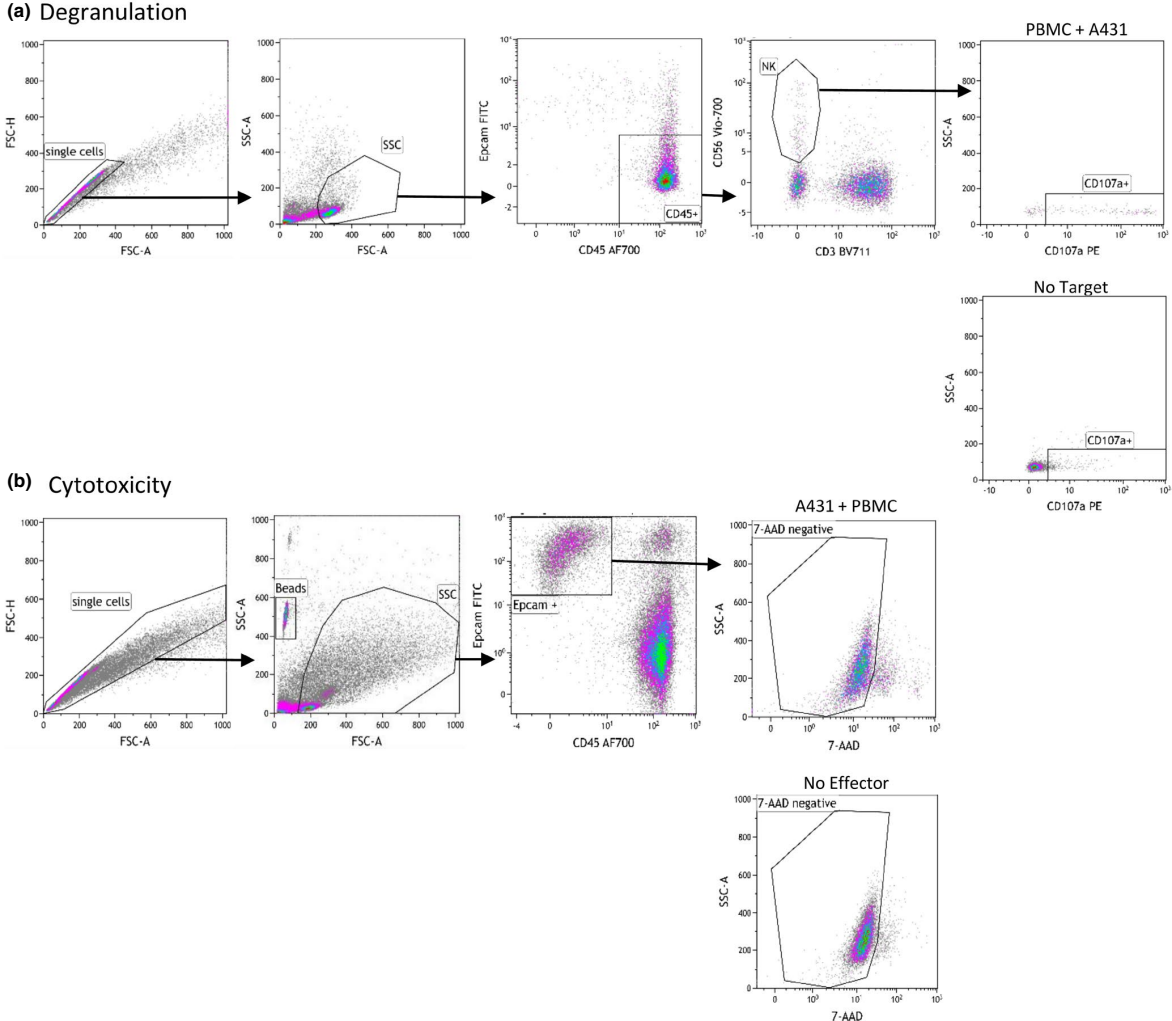
Gating strategy for the degranulation (a) and cytotoxicity (b) assays. Control plot are shown: culture of solely PBMC for the degranulation assay and culture of solely A431 for the cytotoxicity assay

NK cell degranulation and cytotoxicity were analyzed in all blood samples. The assays were performed in the presence and absence of the anti‐epidermal growth factor receptor monoclonal antibody cetuximab (Merck) (EGFR‐mAb) to evaluate variations in NK cell‐mediated antibody‐dependent cell cytotoxicity (ADCC). Peripheral blood mononuclear cells (PBMCs) were isolated from peripheral blood using CPT tubes (BD biosciences) on a density gradient centrifugation. Afterward, isolated PBMC were suspended in Fetal Calf Serum (FCS, Integro), and freezing medium, containing 25% Dimethyl Sulfoxide (DMSO, Sigma Aldrich) and FCS, was added to the cells to reach a final DMSO concentration of 12.5%. Thereafter, the cells were frozen using a controlled rate freezing system and stored in liquid nitrogen. At the time of use, the cells were rapidly thawed and diluted in RPMI (Gibco, Thermo Fischer Scientific) containing 10% FCS, 100 U/ml penicillin, 100 μ/ml streptomycin, 0,3 mg/ml Glutamine (PSG, Gibco, Thermo Fischer Scientific), and 2‐mercaptoethanol (2‐ME, Merck) (details on PBMC viability after thawing are shown in the Supplementary Material). Subsequently, thawed PBMCs underwent a monocyte depletion adherence step (to eliminate the possibility of ADCC by [non‐classical] monocytes) before being activated overnight with 1000 U/ml IL‐2 (Novartis) and 10 ng/ml IL‐15 (ThermoFisher). Next, the monocyte‐depleted PBMC were co‐cultured at an effector to target ratio of 4:1 with the EGFR^+^ A431 (epidermoid carcinoma) cell line that was pre‐incubated for 1 h in the presence or absence of EGFR‐mAb and subsequently washed. The cells were plated in a 96 wells plate in a total volume of 200 μl RPMI for 4 h at 37◦C. NK cell degranulation was based on the percentage of CD107a^+^ (PE, ThermoFisher) NK cells, defined as CD45^+^ (AF700, Biolegend), CD56^+^ (APC‐Vio770, Miltenyi), and CD3^−^ (BV711, BD Horizon). Tumor cell lysis was based on the relative percentage of cytotoxicity [100 ‐ relative percentage of living tumor cells, defined as Epcam^+^ (FITC, Biolegend) and 7AAD^−^ (Sigma Aldrich), quantified with QUANTI BEADS (Invitrogen, Thermo Fisher Scientific)]. Examples of the gating strategy can be found in Figure 1.

### 2.4 NK cell receptor analysis

**FIGURE 2:**
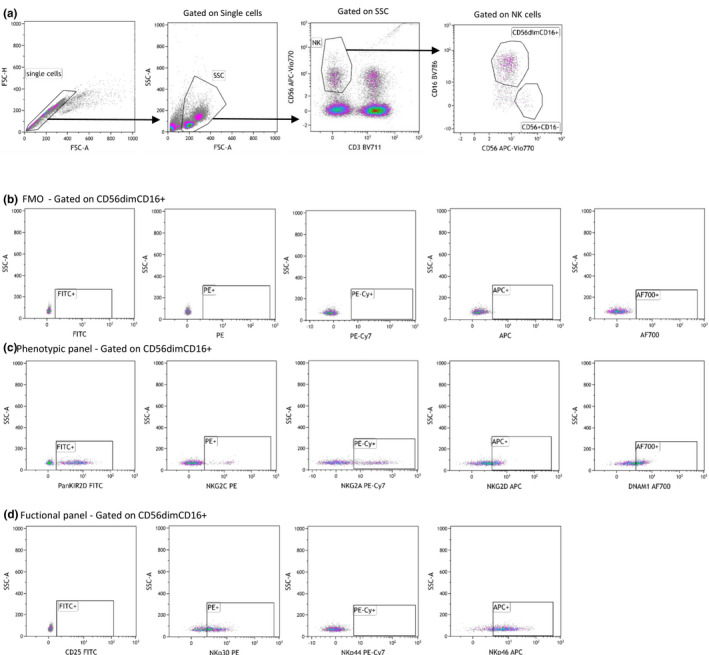
Example of gating strategy for the NK cell receptor analysis. (a) Backbone analysis common to the phenotypical and the functional panel. (b) FMO tube stained with solely the backbone panel used as guidance for the gating of the phenotypical and functional panels. (c) Phenotypical panel which includes PanKIR2D (FITC), NKG2C (PE), NKG2A (PE‐Cy7), NKG2D (APC), and DNAM 1 (AF700). (d) Functional panel which includes CD25 (FITC), NKp30 (PE), NKp44 (PE‐Vio770) and NKp46 (APC)

From a subgroup of patients (4 control and 4 intervention) peripheral blood NK cells were tested for the expression of multiple NK cell receptors using flow cytometry (LSRFortessa™, BD). The following mAbs conjugated with the listed fluorochromes were used for cell staining: PanKIR2D FITC, NKG2C PE, NKG2A PE‐vio770, NKp46 APC, NKp44 PE‐Vio770, CD56 APC‐Vio770 (all from Miltenyi), NKG2D APC (BD Pharmingen), DNAM1 AF700 (RnD systems), NKp30 PE (Biolegend), CD25 FITC, CD3 BV711 (both from BD biosciences) and CD16 BV786 (from BD Horizon). The analyses were performed on both the CD56^dim^CD16^+^ and the CD56^bright^CD16^low/neg^ NK cell subsets. All flow cytometry data were analyzed with the software Kaluza 1.3 (Beckman Coulter). Examples of the gating strategy are shown in Figure 2. Details on the catalog and clone numbers of all antibodies used can be found in Table S1.

### 2.5 IL‐6 analysis

At T0 and T1, plasma IL‐6 levels were assessed via an enzyme‐linked immunosorbent assay (ELISA, R&D systems).

### 2.6 Statistical analysis

Within‐group and between‐group differences in NK cell activity and IL‐6 levels were examined with linear regression analyses. In the models, the posttest value was the dependent variable. When examining within‐group changes, we included the baseline as independent variable in the model for the study arms separately. To examine between‐group differences, the intervention was included as independent variable and the models were adjusted for the baseline value of the study arm. Unstandardized regression coefficients, representing the average change in the dependent variable over time within each group or the difference between exercise and control group, and corresponding 95% confidence intervals (CI) were presented. Due to the small sample size and not normal distribution of the NK receptor results, a Mann‐Whitney U test was used to examine the difference between groups in the changes from baseline to posttest. Median and interquartile range (IQR) were shown. Additionally, due to the small sample size, the interpretation of the results was based on potentially relevant differences (≥10% group differences), instead of solely the *p*‐value (Panagiotakos, 2008; Prel et al., 2009). All analyses were conducted using SPSS (IBM SPSS Statistics 22).

## 3 RESULTS

Of the 30 patients enrolled, 22 completed the follow‐up assessments. We were able to analyze blood samples of 14 of these patients (8 exercise; 6 control). Patient characteristics are shown in Table 1.

**TABLE 1:** Demographic and clinical characteristics of patients who completed this study (*n* = 14)

	Exercise intervention (*n* = 8)	Control (*n* = 6)
Age, mean (SD) years	55.1 (14.8)	60.7 (7.6)
Gender, *n* (%) female	6 (75)	6 (100)
BMI, mean (SD) kg/m**^2^**	25.8 (6.9)	28.7 (4.5)
Marital status. *n* (%) married	5 (62.5)	4 (66.7)
Education level, *n* (%) high	3 (37.5)	2 (33.3)
Employment status, *n* (%) working	5 (62.5)	3 (50.0)
Smoking, *n* (%) yes	0 (0)	0 (0)
Exercise history, *n* (%) yes	6 (75.0)	5 (83.3)
Co‐morbidities, * *n* (%) yes	3 (37.5)	4 (66.7)
Cancer type and treatment, *n* (%)		
Breast cancer, Adjuvant	2 (25)	3 (50)
Breast cancer, Neoadjuvant	2 (25)	1 (17)
Colon cancer, Adjuvant	4 (50)	2 (33)

Abbreviations: BMI, body mass index; SD, standard deviation.

* Any of the following comorbidities: cardiovascular disease, high blood pressure, osteoporosis, asthma, neurological disease, gastrointestinal disease, psychiatric problems, degenerative disease, and migraine.

**FIGURE 3:**
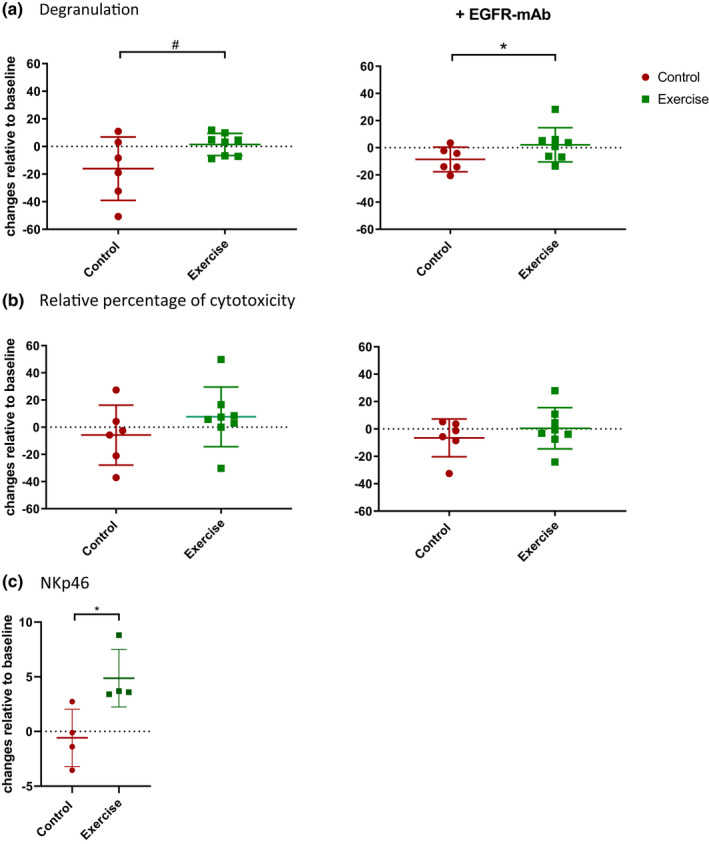
(a) Changes from baseline of pbNK cell degranulation after 4h co‐culture with A431 in presence and absence of EGFR‐mAb. E:T ratio 4:1. *N* = 8 exercise cohort (green) and *N* = 6 control cohort (red). The bars represent the mean ±SD. (b) Changes from baseline of the relative percentage of cytotoxicity after 4h A431 and PBMC co‐culture in presence and absence of EGFR‐mAB. E:T ratio 4:1. *N* = 8 exercise cohort (green) and *N* = 6 control cohort (red). The bars represent the mean ±SD. (c) NKp46 expression on the CD56dim CD16pos NK subset: median fluorescence index (MFI) changes from baseline. *N* = 4 exercise group and *N* = 4 control group. The bars represent median ±IQR. Significance is presented as ^#^ *p* < 0.1, *p < 0.05

NK cell degranulation after (neo)adjuvant chemotherapy was preserved in the exercise group whereas it decreased in the control group, resulting in a between‐group difference of 13.8% (95%CI = −2.5;30.0, *p* = 0.09), in favor of the exercise group (Table 2; Figure 3a). A similar result was found when PBMCs were co‐cultured with EGFR‐mAb coated A431 cells (*β* = 11.4%, 95%CI = 0.57;22.3, *p* = 0.04).

**TABLE 2:** NK cell degranulation, CD56^dim^CD16^pos^ NK cells phenotype, lysis of A431 tumor cells and IL‐6 plasma levels at baseline and at 9–12 weeks and changes within and between the exercise intervention and control group

Variable	*N*	Baseline	9–12 weeks	Within‐Group change	Between‐Group change	
		Mean (SD)	Mean (SD)	β (95% CI)	β (95% CI)	*p* value
%CD107a^pos^ NK cells						
A431 vs PBMC						
Exercise	8	34.7 (19.6)	36.1 (20.4)	1.4 (−5.3; 8.2)	13.8 (−2.5; 30.0)	0.09
Control	6	42.7 (22.2)	26.6 (10.1)	−16.1 (−40.2; 8.0)		
+ EGFR‐mAb						
Exercise	8	65.4 (17.6)	67.5 (14.4)	2.2 (−8.3; 12.7)	11.4 (0.6; 22.3)	0.04
Control	6	63.8 (14.3)	55.2 (9.8)	−8.6 (−18.1; 0.9)		
Relative Percentage of Cytotoxicity						
A431 vs PBMC						
Exercise	8	41.5 (28.1)	49.1 (21.1)	7.6 (−10.8; 26.0)	13.7 (10.1; 37.5)	0.23
Control	6	40.8 (28.7)	35.0 (31.5)	−5.8 (−28.9; 17.3)		
+ EGFR‐mAb						
Exercise	8	62.1 (29.6)	62.6 (24.2)	0.5 (−12.1; 13.1)	7.4 (9.1; 23.9)	0.34
Control	6	60.4 (18.1)	53.9 (22.9)	−6.5 (−21.0; 8.0)		
IL−6 (pg/ml)						
Exercise	8	0.2 (0.1)	0.5 (0.2)	0.3 (0.1; 0.5)	0.2 (−0.1; 0.5)	0.10
Control	6	0.3 (0.1)	0.4 (0.2)	0.1 (−0.2; 0.3)		

CD56^dim^CD16^pos^ NK cells – Median Fluorescence Intensity					
Variable	*N*	Baseline	9–12 weeks	Within‐group change	Between‐group change
		Median (IQR)	Median (IQR)	Median (IQR)	*p*‐value
KIR2D					
Exercise	4	4.9 (1.8;5.7)	3.0 (0.7;6.6)	−0.4 (−3.2;1.4)	0.686
Control	4	3.2 (0.9;4.1)	2.6 (0.3;4.5)	0.1 (−1.8;1.0)	
NKG2A					
Exercise	4	3.4 (2.4;16.2)	8.9 (4.2;22.9)	4.7 (1.5;7.6)	0.486
Control	4	3.0 (2.1;4.6)	3.6 (2.3;25.6)	0.6 (−0.2;21.3)	
DNAM1					
Exercise	4	2.1 (2.0;3.0)	2.0 (2.0;2.1)	−0.1 (−1.0;0.02)	0.886
Control	4	1.9 (1.8;2.7)	1.9 (1.8;2.4)	−0.03 (−0.3;0.1)	
NKG2C					
Exercise	4	0.6 (0.4;4.9)	0.8 (0.7;1.3)	0.2 (−3.6;0.3)	0.686
Control	4	0.4 (0.3;0.5)	0.6 (0.4;0.9)	0.2 (0.1;0.4)	
NKG2D					
Exercise	4	1.3 (1.15;1.6)	2.8 (2.7;3.0)	1.4 (1.1;1.8)	0.343
Control	4	1.5 (1.3;1.8)	2.3 (1.9;3.5)	0.6 (0.4;2.1)	
NKp30					
Exercise	4	2.1 (1.3;3.2)	3.5 (2.5;5.2)	1.1 (0.1;3.4)	0.686
Control	4	3.0 (1.5;4.2)	3.8 (1.6;5.2)	0.3 (−0.1;1.7)	
NKp44					
Exercise	4	0.5 (0.4;0.6)	0.2 (−0.13;0.3)	−0.2 (−0.7;−0.1)	0.114
Control	4	0.3 (0.2;0.6)	0.6 (0.5;0.7)	0.3 (0.0;0.4)	
NKp46					
Exercise	4	4.7 (4.0;8.5)	10.6 (8.3;13.0)	3.6 (3.4;7.5)	0.029
Control	4	5.8 (3.6;8.1)	5.1 (2.7;7.9)	−0.7 (−3.0;2.0)	

Abbreviations: EGFR, epidermal growth factor receptor; IQR, interquartile range; mAb, monoclonal antibody; MFI, median fluorescence index; PBMC, Peripheral blood mononuclear cell.

The relative percentages of cytotoxicity showed an average increase in the exercise group and decrease in the control group (Table 2; Figure 3b), resulting in a (non‐significant) between‐group difference of 7.4%, (95%CI = −9.1; 23.9, *p *= 0.34) in the presence and 13.7%, (95%CI = −10.1;37.5, *p* = 0.23) in the absence of EGFR‐mAb.

A significantly higher expression of the activating receptor NKp46 was found on CD56^dim^CD16^+^ NK cells in the exercise group compared to the control group with a median change of respectively 3.6 (IQR:3.4;7.5) and −0.7 (IQR:‐3.0;2.0) in median fluorescence intensity (Table 2; Figure 3c). No additional relevant differences in expression were found for the other NK cell receptors on the CD56^dim^CD16^+^ NK cells (Table 2) as well as on the CD56^bright^CD16^low/neg^ NK cells (Table S2).

Finally, though not statistically significant, plasma IL‐6 levels were slightly increased in the exercise group compared to the control group (*β* = 0.2 pg/ml, 95%CI = −0.1;0.5; *p* = 0.10) (Table 2).

## 4 DISCUSSION

In this randomized controlled pilot trial, we evaluated the effect of a combined resistance and aerobic exercise intervention on NK cell phenotype and function in patients with breast or colon cancer undergoing (neo)adjuvant chemotherapy. Due to the small sample size and the explorative nature of this pilot study, the results should be viewed more as hypothesis‐generating than hypothesis‐testing. The results suggest that exercise may preserve NK cell activity indicated by preserved degranulation levels upon exposure to tumor cells and increased expression of the activating receptor NKp46 on effector NK cells (Cooper et al., 2001). Moreover, an average between‐group difference of 13.7% in PBMC mediated cytotoxicity in favor of the exercise group was found suggesting that it is worth conducting a future trial investigating the effect of exercise during chemotherapy on PBMC mediated cytotoxicity. The results of this study could be used to inform sample size calculations of such a trial. In line, an average between‐group difference of 7.4% was observed when tumor cells were exposed to an EGFR‐mAb. Finally, the IL‐6 levels were found slightly increased during exercise, which aligns with published data from mouse models, and may be related to NK cell re‐distribution to the tumor microenvironment (Pedersen et al., 2016). Nevertheless, larger differences would likely be found when analyzing the acute exercise‐dependent IL‐6 release. It is currently unclear how the approximate 10% difference in NK cell activity translates to clinical outcomes. However, higher NK cell activity has been associated with better response to neoadjuvant chemotherapy (Kim et al., 2019) and antibody‐based therapies (Beano et al., 2008; Liljefors et al., 2003) suggesting that combining cancer therapies with exercise might be beneficial. Moreover, previous studies showed that the expression of NKp46 was positively associated with overall survival in patients with metastatic prostate cancer (Pasero et al., 2015) and with acute myeloid leukemia (Fauriat et al., 2007). The considerable interest in exercise by both groups was reflected in the high proportion of history in sport participation consequently our study sample may be somewhat biased towards a more active cancer population. We were unable to rule out potential contamination resulting from exercising patients in the control group, which might have underestimated the effect of the intervention on NK cell activity. However, we aimed to limit potential contamination by offering the exercise intervention to the control group in the second half of the chemotherapy, and offering supervised moderate‐high intensity exercise which may be more effective compared to home‐based low‐intensity (Waart et al., 2015). In conclusion, moderate‐high intensity resistance and aerobic exercise during chemotherapy has the potential to maintain NK cell function. For definite conclusions, findings should be replicated in a study powered for this purpose.

## CONFLICT OF INTEREST

J.J. vd Vliet is chief scientific officer at Lava Therapeutics. Further no other conflict of interest.

## AUTHOR’S CONTRIBUTION

Altenburg, Buffart, and Verheul conceived and designed the Metric trial; Altenburg, Buffart, Sweegers, and Verheul coordinated the METRIC trial; Buffart, de Gruijl, Toffoli, and vd Vliet conceived and designed the NK analysis; Bontkes and Toffoli performed the experiments; Buffart and Toffoli analyzed the data; Buffart, de Gruijl, Toffoli, and vd Vliet interpretated the results; Buffart and Toffoli drafted the manuscript and prepared tables and figures; all the authors edited the manuscript and approved the final version.

## Supporting information



Table S1‐S2
