# Supplementary material for: Effects of physical exercise on natural killer cell activity during (neo)adjuvant chemotherapy: A randomized pilot study
Source: Physiol Rep. 2021 Jun 10;9(11):e14919. doi: 10.14814/phy2.14919 (PMC8191403; doi:10.14814/phy2.14919)
Supplement: Supplementary file 1 — Table S1‐S2 [file PHY2-9-e14919-s001.docx]

*Supplementary material: analysis of PBMC viability after the freezing and thawing process*

An analysis of PBMC viability after the freezing and thawing process was conducted on samples from a subgroup of patients (N=6), from both the exercise and control groups, collected after the end of the chemotherapy. Viability was assessed as percentage 7AAD^-^ cells.

A mean PBMC viability of 98.1 (SD: 0.9) was found. Moreover, the analysis of NK cell viability showed a mean percentage of 7AAD^-^ cells of 98.0 (SD: 3.2) and 99.8 (SD: 0.5) for the CD56^dim^CD16^+^ and the CD56^bright^CD16^-^ NK cells respectively.

*Supplementary Table 1: catalogue numbers of used antibodies*

| Antibodies | Brand | Catalog number | Clone |
| --- | --- | --- | --- |
| CD107a PE | ThermoFisher | 12-1079-42 | eBioH4A3 |
| CD45 AF700 | Biolegend | 304024 | HI30 |
| CD56 APC-Vio770 | Miltenyi | 130-114-548 | REA196 |
| CD3 BV711 | BD Horizon | 563725 | UCHT1 |
| Epcam FITC | Biolegend | 324204 | 9C4 |
| PanKIR2D FITC | Miltenyi | 130-098-689 | NKVFS1 |
| NKG2C PE | Miltenyi | 130-119-776 | REA205 |
| NKG2A PE-vio770 | Miltenyi | 130-114-093 | REA110 |
| NKp46 APC | Miltenyi | 130-099-092 | 9E2 |
| NKp44 PE-Vio770 | Miltenyi | 130-120-626 | 2.29 |
| NKG2D APC | BD Pharmingen | 558071 | 1D11 |
| DNAM1 AF700 | RnD systems | FAB666N-100UG | 102511 |
| NKp30 PE | Biolegend | 325207 | P30-15 |
| CD25 FITC | BD biosciences | 345796 | 2A3 |
| CD16 BV786 | BD Horizon | 563690 | 3G8 |

*Supplementary Table 2: CD56^bright^CD16^low/neg^ NK cells* *phenotype at baseline and at 9-12 weeks and changes within and between the exercise intervention and control group*

| CD56^bright^CD16^low/neg^ NK cells – Median Fluorescence Intensity | | | | | |
| --- | --- | --- | --- | --- | --- |
| Variable | **N** | **Baseline** | **9-12 weeks** | **Within-group change** | **Between-group change** |
|  |  | Median (IQR) | Median (IQR) | Median (IQR) | P-value |
| KIR2D  Exercise  Control | 4  4 | 0.01 (-0.03;0.05)  0.05 (0.04;0.1) | 0.1 (0.1;0.2)  0.1 (0.1;0.1) | 0.1 (0.1;0.2)  0.03 (0.005;0.06) | 0.057 |
| NKG2A  Exercise  Control | 4  4 | 73.9 (46.6;85.6)  61.0 (54.1;66.4) | 78.0 (65.2;84.2)  4.6 (63.5;96.6) | 7.1 (-4.2;18.6)  15.6 (7.3;30.2) | 0.343 |
| DNAM1  Exercise  Control | 4  4 | 1.2 (0.9;2.2)  0.6 (0.4;1.2) | 2.295 (1.9225;2.63)  1.92 (1.665;2.61) | 1.0 (-0.3;1.8)  1.5 (0.6;1.9) | 0.686 |
| NKG2C  Exercise  Control | 4  4 | 1.4 (1.0;2.4)  1.1 (0.8;1.3) | 1.5 (1.3;1.6)  1.3 (1.1;1.7) | 0.1 (-0.8;0.3)  0.2 (0.1;0.4) | 0.343 |
| NKG2D  Exercise  Control | 4  4 | -0.2 (-0.9;0.6)  -0.4 (-1.5;1.4) | 3.8 (3.3;4.5)  3.1 (2.4;4.5) | 4.0 (3.5;4.6)  3.5 (1.8;5.2) | 0.686 |
| NKp30  Exercise  Control | 4  4 | 2.4 (2.3;3.2)  2.3 (1.9;2.5) | 3.2 (2.9;3.4)  2.5 (2.0;3.2) | 0.6 (0.1;0.8)  0.3 (-0.2;0.8) | 0.686 |
| NKp44  Exercise  Control | 4  4 | 0.5 (0.2;0.7)  0.4 (0.3;0.9) | 0.6 (0.2;0.9)  0.9 (0.7;1.2) | 0.1 (-0.5;0.8)  0.4 (0.1;0.6) | 0.686 |
| NKp46  Exercise  Control | 4  4 | 24.8 (17.9;35.7)  22.2 (21.2;29.5) | 32.5 (28.6;35.8)  23.2 (12.6;28.5) | 7.6 (0.1;10.8)  -3.2 (-9.6;4.3) | 0.200 |
